# Association of LDL:HDL ratio with prediabetes risk: a longitudinal observational study based on Chinese adults

**DOI:** 10.1186/s12944-022-01655-5

**Published:** 2022-05-15

**Authors:** Maobin Kuang, Nan Peng, Jiajun Qiu, Yanjia Zhong, Yang Zou, Guotai Sheng

**Affiliations:** 1 Cardiology Department, Jiangxi Provincial People’s Hospital, Nanchang, 330006 China; 2 Endocrinology Department, Jiangxi Provincial People’s Hospital, Nanchang, 330006 China; 3 Jiangxi Cardiovascular Research Institute, Jiangxi Provincial People’s Hospital, Nanchang, 330006 China

**Keywords:** LDL:HDL ratio, Prediabetes, Chinese adults, Longitudinal cohort

## Abstract

**Background:**

Low-density lipoprotein:high-density lipoprotein cholesterol ratio (LDL:HDL ratio) has a good performance in identifying diabetes mellitus (DM) and insulin resistance. However, it is not yet clear whether the LDL:HDL ratio is associated with a high-risk state of prediabetes.

**Methods:**

This cohort study retrospectively analyzed the data of 100,309 Chinese adults with normoglycemia at baseline. The outcome event of interest was new-onset prediabetes. Using multivariate Cox regression and smoothing splines to assess the association of LDL:HDL ratio with prediabetes.

**Results:**

During an average observation period of 37.4 months, 12,352 (12.31%) subjects were newly diagnosed with prediabetes. After adequate adjustment for important risk factors, the LDL:HDL ratio was positively correlated with the prediabetes risk, and the sensitivity analysis further suggested the robustness of the results. Additionally, in stratified analysis, we discovered significant interactions between LDL:HDL ratio and family history of DM, sex, body mass index and age (all *P*-interaction < 0.05); among them, the LDL:HDL ratio-related prediabetes risk decreased with the growth of body mass index and age, and increased significantly in women and people with a family history of DM.

**Conclusions:**

The increased LDL:HDL ratio in the Chinese population indicates an increased risk of developing prediabetes, especially in women, those with a family history of DM, younger adults, and non-obese individuals.

**Supplementary Information:**

The online version contains supplementary material available at 10.1186/s12944-022-01655-5.

## Background

Diabetes mellitus (DM) is a metabolic disorder syndrome caused by genetic and environmental factors, characterized by decreased insulin sensitivity, insulin deficiency, and impaired biological function [1]. DM has become a serious health problem worldwide due to its high prevalence and associated disability and mortality [2, 3].

Prediabetes refers to a pathological stage in which blood glucose concentration is between normal blood glucose levels and DM. It is a high-risk factor for developing DM and is also a significant factor for retinopathy, chronic kidney disease and cardiovascular disease [4]. Recent epidemiological studies have shown that according to the criteria of the American Diabetes Association’s (ADA’s) diagnostic, the prevalence of prediabetes is approximately 35.7% in China [5], which is significantly higher than that of other chronic diseases. It is estimated that about 5–10% of prediabetic patients will progress to DM every year, and the proportion of patients who eventually develop DM exceeds 70% [6]. Furthermore, the Diabetes Prevention Program Research Group also pointed out that lifestyle interventions can reduce the development of DM by 58% in prediabetic patients [7]. Therefore, early diagnoses and interventions of prediabetes are very important to prevent DM.

Prediabetes is characterized by impaired glucose tolerance or impaired fasting glucose (IFG). However, there are currently no unified diagnostic criteria for prediabetes [8]. The diagnostic criteria for prediabetes defined by the ADA are widely adopted in China, that is, the IFG threshold is defined as fasting plasma glucose (FPG): 5.6–6.9 mmol/L [9]. Insulin resistance (IR) is currently recognized as a vital pathophysiological mechanism for the occurrence and development of prediabetes. Observational studies have further confirmed that IR is an important marker for the development of prediabetes from normal blood glucose levels in healthy people [10, 11]. Therefore, measuring IR is a valid method for assessing prediabetes risk. However, the current measurement of IR is too complex to be suitable for large-scale population screening [12, 13], and it may be more suitable to find effective IR surrogate markers for assessing the risk of prediabetes. Recent studies have pointed out that the low-density lipoprotein:high-density lipoprotein cholesterol ratio (LDL:HDL ratio) has high accuracy in identifying IR and is regarded as a potentially valuable surrogate marker for IR [14, 15]. In further studies, more scholars have found that the LDL:HDL ratio also has some value in diseases risk assessment such as DM, nonalcoholic fatty liver disease (NAFLD) and carotid plaque formation in male patients with type 2 DM [16–18]. These findings all indicated that the LDL:HDL ratio could be a good marker for blood glucose metabolism. Nonetheless, the relationship of LDL:HDL ratio with prediabetes is currently unclear. Therefore, this study retrospectively analyzed the association of LDL:HDL ratio with prediabetes based on a large Chinese adult population.

## Methods

### Study subjects and design

**Fig. 1:**
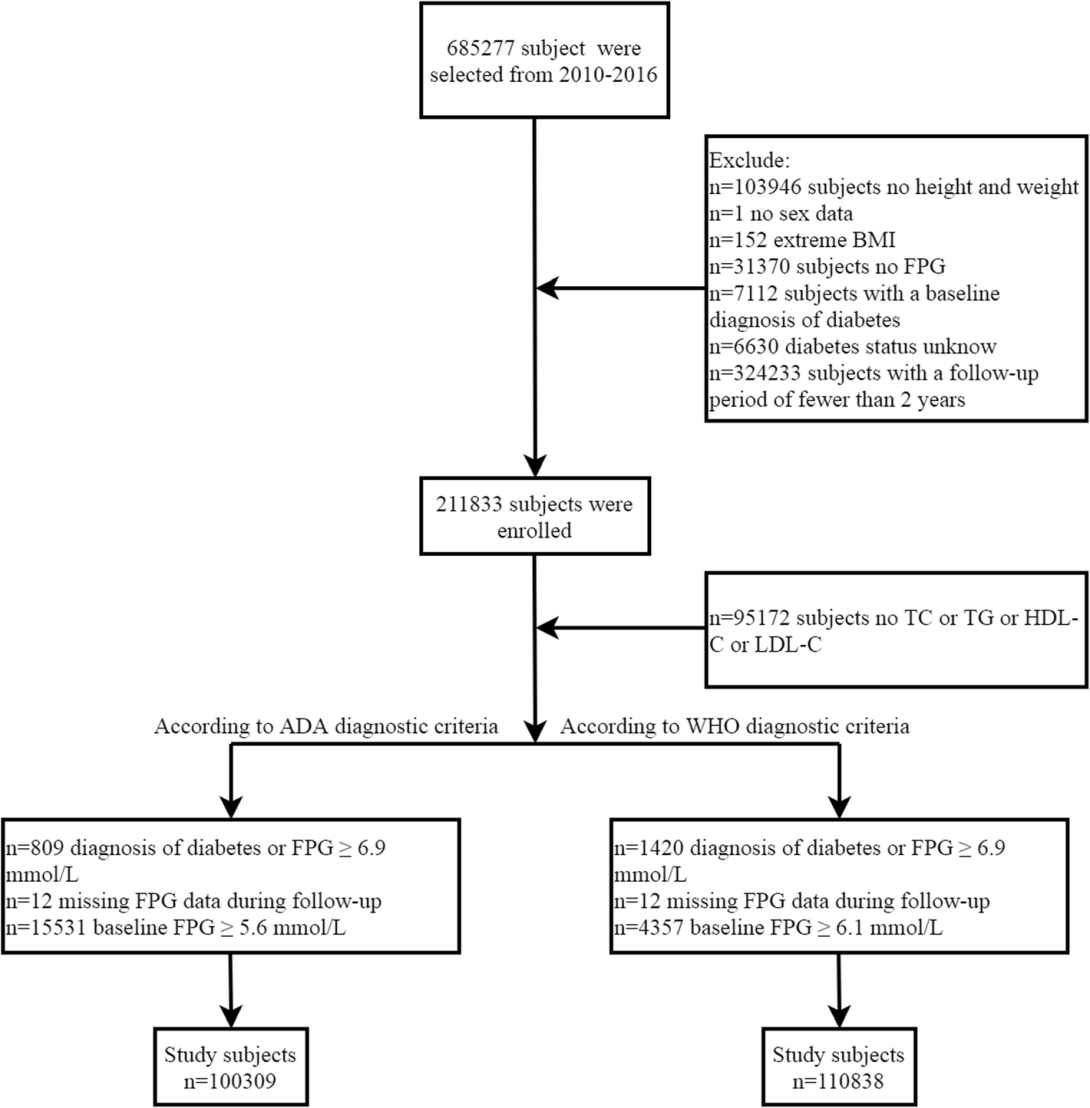
Flow diagram of subjects included in the cohort study

The data of this study were obtained from the Dryad (www.Datadryad.org) public database, and the original data were provided by professor Li [19]. This dataset collects all medical data of Chinese adults over the age of 20 (*n* = 685,227) who underwent health checks at Rich Healthcare Group in China from 2010 to 2016. Referring to the articles of service of the Dryad database, the dataset can be used by researchers to conduct secondary analyses [19]. In a previous study by Li et al. [20], they excluded data from the dataset of subjects with missing values, extreme body mass index (BMI) values, a follow-up interval of less than 2 years, DM diagnosed at baseline, and subjects with uncertain DM status during follow-up. Ultimately, data of 211,833 subjects were enrolled in their study and analyzed. Our present study performed a secondary analysis using the same dataset as Li et al., intending to explore the association of LDL:HDL ratio with prediabetes risk. Subjects with the following characteristics were further excluded from this study according to the ADA’s or World Health Organization’s (WHO’s) diagnostic criteria for prediabetes [9, 21]: (1) FPG ≥ 5.6 mmol/L (ADA; *n* = 15,531) or FPG ≥ 6.1 mmol/L (WHO; *n* = 4357) at baseline; (2) missing data on lipid-related parameters (*n* = 95,172); (3) loss of FPG data during follow-up (*n* = 12); and (4) self-reported DM diagnosed by an endocrinologist or FPG ≥ 6.9 mmol/L in the follow-up period (ADA: *n* = 809; WHO: *n* = 1420). Finally, we included 100,309 and 110,838 subjects based on ADA’s and WHO’s diagnostic criteria for prediabetes, respectively (Fig. 1). The main results in our present study were analyzed using the cohort data determined by the ADA’s diagnostic criteria, and the cohort data determined by the WHO’s diagnostic criteria was used as a sensitivity analysis to verify the robustness of the results. Owing to the ethics committee of Rich Healthcare Group having approved the previous research, repeated application of the current study for ethical approval and informed consent were exempted by the ethics committee of People’s Hospital of Jiangxi Provincial (ethical review No. 2021–067).

### Clinical index measurement

A detailed questionnaire containing sociodemographic information such as age, sex, smoking and drinking habits, and family history of DM was completed by the subjects at each visit to the health screening center. The height, weight, and blood pressure of the subjects were measured by the physical examiner. Height and weight were measured with subjects standing and wearing light clothing and no shoes to the nearest 0.1 cm and 0.1 kg, respectively. A mercury sphygmomanometer was used to measure the subjects’ blood pressure at calm. BMI was calculated as weight/height^2^.

After the subjects fasting for at least 10 hours, venous blood samples were drawn by professional medical workers, and the Beckman 5800 automatic analyzer was used to determine the total cholesterol (TC), alanine aminotransferase (ALT), triglyceride (TG), creatinine (Cr), FPG, high-density lipoprotein cholesterol (HDL-C), aspartate aminotransferase (AST), blood urea nitrogen (BUN) and low-density lipoprotein cholesterol (LDL-C) levels; among which, the level of FPG was measured by glucose oxidase method, while the concentrations of other blood lipid parameters and biochemical indexes were measured by optical turbidimetry.

### Diagnosis of prediabetes

According to one of the ADA’s 2018 diagnostic criteria for prediabetes, IFG was used as the basis for the diagnosis of prediabetes, and the FPG values in prediabetic patients were set at 5.6 to 6.9 mmol/L [9]; similarly, the WHO set FPG levels in prediabetic patients to 6.1 to 6.9 mmol/L [21].

### Statistical analysis

Data analysis of this study was done with R language (version 3.4.3) and Empower (R) (version 2.20). *P*-value of < 0.05 (bilateral) was considered statistically significant. We analyzed the data by the following steps to test the research hypothesis.

First, we described the baseline information of subjects by quartiles of the LDL:HDL ratio. Categorical variables were denoted by frequency (percentage). QQ plot and Kolmogorov-Smirnov test were used to verify the distribution type of continuous variables, in which the normally distributed variables were represented by the average (standard deviation) and the skewed distributed variables were represented by the median (interquartile range). Choose Kruskal-Wallis H test or Chi-Square test or one-way ANOVA test for comparison between groups according to the type of variables and its distribution.

Second, three multivariate Cox regression models and Kaplan-Meier curves were established and we recorded the hazard ratio (HR) and 95% confidence interval (CI) related to LDL:HDL ratio and the risk of prediabetes. Firstly, we evaluated a crude model that had not been adjusted and regarded it as a reference model for other models. To determine the influence of blood glucose and lipids on prediabetes, we considered FPG, TC, TG, and HDL-C as confounding factors in Model 1. Model 2 included variables from model 1 plus sex, BMI and age. Finally, to further assess the independent relationship of the LDL:HDL ratio with prediabetes, we adjusted non-collinear variables with > 10% effect on the prediabetes risk associated with the LDL:HDL ratio in Model 3. Moreover, we attempted to use smoothing splines for curve fitting based on Model 3 to gain insight into the shape of the association of LDL:HDL ratio with the incidence of prediabetes.

Third, we performed several sensitivity analyses, removing subjects with a family history of DM, abnormal blood pressure, and normal BMI values. We also conducted the same analyses by including subjects based on the WHO’s diagnostic criteria for prediabetes [21].

Fourth, to explore other risk factors affecting the association of LDL:HDL ratio with the incidence of prediabetes, this study also conducted several exploratory analyses based on model 3 which were stratified by family history of DM, BMI, TG, age, and sex. Likelihood ratio tests were conducted to demonstrate whether there were indeed differences between subgroups.

Fifth, we also constructed receiver operating characteristic curves to estimate the predictive power of the LDL:HDL ratio and its individual components, LDL-C and HDL-C, for the risk of prediabetes, and compared the area under the receiver operating characteristic curve (AUROC) of the three using the Delong test to explore the best predictor of prediabetes risk.

## Result

### Baseline characteristics of subjects

We included 100,309 subjects (mean age: 42.9 years old, 51.97% man, 48.03% women) with normoglycemia at baseline according to the ADA’s diagnostic criteria for prediabetes. Table 1 shows the baseline characteristics of the subjects based on the quartiles of the LDL:HDL ratio. Subjects with a higher LDL:HDL ratio were usually male, older and showed higher diastolic blood pressure (DBP), height, FPG, TC, TG, AST, weight, ALT, BMI, LDL-C, systolic blood pressure (SBP), BUN and Cr levels, but lower HDL-C concentrations (all *P* < 0.05). Additionally, the proportion of subjects with a family history of DM was not statistically different between quartiles of LDL:HDL ratio (*P* = 0.659).

**Table 1:** Baseline characteristics of subjects according to the LDL:HDL ratio quartiles

	LDL:HDL ratio quartiles				
	Q1 (0.01–1.61)	Q2 (1.61–1.95)	Q3 (1.96–2.41)	Q4 (2.41–19.11)	*P*-value
No. of subjects	25,077	25,075	25,069	25,088	
Sex					< 0.001
Men	8843 (35.26%)	11,561 (46.11%)	14,463 (57.69%)	17,263 (68.81%)	
Women	16,234 (64.74%)	13,514 (53.89%)	10,606 (42.31%)	7825 (31.19%)	
Age, years	36.00 (32.00–45.00)	39.00 (33.00–49.00)	41.00 (34.00–52.00)	44.00 (35.00–56.00)	< 0.001
Height, cm	164.64 (7.82)	165.54 (8.26)	166.74 (8.38)	168.02 (8.33)	< 0.001
Weight, kg	58.78 (10.30)	62.42 (11.13)	65.84 (11.57)	69.50 (11.86)	< 0.001
BMI, kg/m^2^	21.60 (2.87)	22.69 (3.07)	23.58 (3.09)	24.52 (3.09)	< 0.001
SBP, mmHg	114.01 (15.04)	116.88 (15.78)	119.60 (16.17)	121.81 (16.24)	< 0.001
DBP, mmHg	71.00 (10.14)	72.85 (10.55)	74.63 (10.71)	76.55 (10.86)	< 0.001
FPG, mmol/L	4.76 (4.42–5.06)	4.86 (4.52–5.14)	4.88 (4.53–5.17)	4.88 (4.52–5.20)	< 0.001
TC, mmol/L	4.23 (0.71)	4.53 (0.71)	4.84 (0.76)	5.39 (0.89)	< 0.001
TG, mmol/L	0.76 (0.58–1.04)	0.93 (0.70–1.30)	1.18 (0.85–1.64)	1.54 (1.11–2.14)	< 0.001
HDL-C, mmol/L	1.62 (0.30)	1.44 (0.24)	1.33 (0.23)	1.14 (0.22)	< 0.001
LDL-C, mmol/L	2.19 (0.44)	2.57 (0.43)	2.86 (0.49)	3.36 (0.66)	< 0.001
ALT, U/L	14.50 (11.00–20.60)	16.00 (12.00–23.90)	18.90 (13.60–28.00)	22.50 (15.90–34.00)	< 0.001
AST, U/L	20.30 (17.40–24.10)	21.00 (18.00–25.20)	22.00 (19.00–27.00)	23.10 (19.90–28.00)	< 0.001
BUN, mmol/L	4.44 (1.13)	4.63 (1.15)	4.70 (1.16)	4.77 (1.16)	< 0.001
Cr, umol/L	64.92 (14.94)	68.81 (15.29)	72.01 (15.65)	73.89 (15.49)	< 0.001
Family history of diabetes					
	546 (2.18%)	572 (2.28%)	532 (2.12%)	558 (2.22%)	0.659
Smoking status					< 0.001
No	717 (2.86%)	1000 (3.99%)	1423 (5.68%)	2209 (8.81%)	
Past	177 (0.71%)	215 (0.86%)	303 (1.21%)	395 (1.57%)	
Current	5858 (23.36%)	5097 (20.33%)	5021 (20.03%)	5223 (20.82%)	
Not recorded	18,325 (73.07%)	18,763 (74.83%)	18,322 (73.09%)	17,261 (68.80%)	
Drinking status					< 0.001
No	142 (0.57%)	141 (0.56%)	152 (0.61%)	204 (0.81%)	
Past	878 (3.50%)	1038 (4.14%)	1286 (5.13%)	1346 (5.37%)	
Current	5732 (22.86%)	5133 (20.47%)	5309 (21.18%)	6277 (25.02%)	
Not recorded	18,325 (73.07%)	18,763 (74.83%)	18,322 (73.09%)	17,261 (68.80%)	

*Abbreviations*: *BMI* body mass index, *SBP* systolic blood pressure, *DBP* diastolic blood pressure, *FPG* fasting plasma glucose, *TG* triglyceride, *TC* total cholesterol, *HDL-C* high-density lipoprotein cholesterol, *LDL-C* low-density lipoprotein cholesterol, *ALT* alanine aminotransferase, *AST* aspartate aminotransferase, *BUN* blood urea nitrogen, *Cr* creatinine, *LDL:HDL ratio* low-density lipoprotein:high-density lipoprotein cholesterol ratio

### The incidence of prediabetes

**Fig. 2:**
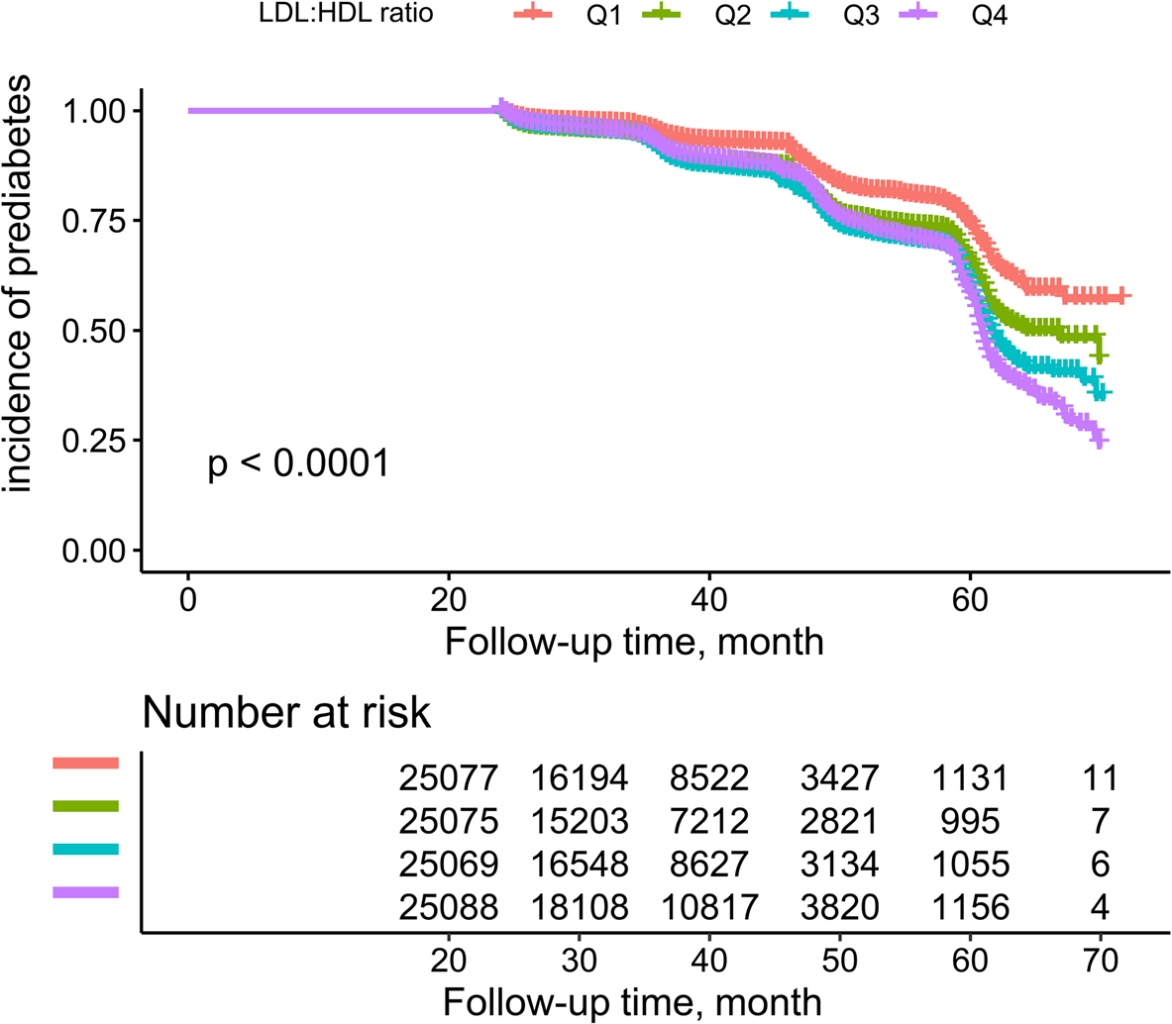
Kaplan-Meier analysis of future prediabetes risk according to LDL:HDL ratio quartiles. LDL:HDL ratio: low-density lipoprotein:high-density lipoprotein cholesterol ratio

During an average observation period of 37.4 months, a total of 12,352 (12.31%) subjects were diagnosed with new-onset prediabetes. Figure 2 shows the trend of cumulative incidence of prediabetes which was grouped by the quartiles of LDL:HDL ratio with follow-up time. We found that there was an increasing cumulative incidence of prediabetes with the increasing LDL:HDL ratio quartiles (log-rank *P* < 0.001).

### Association of LDL:HDL ratio with prediabetes risk

**Fig. 3:**
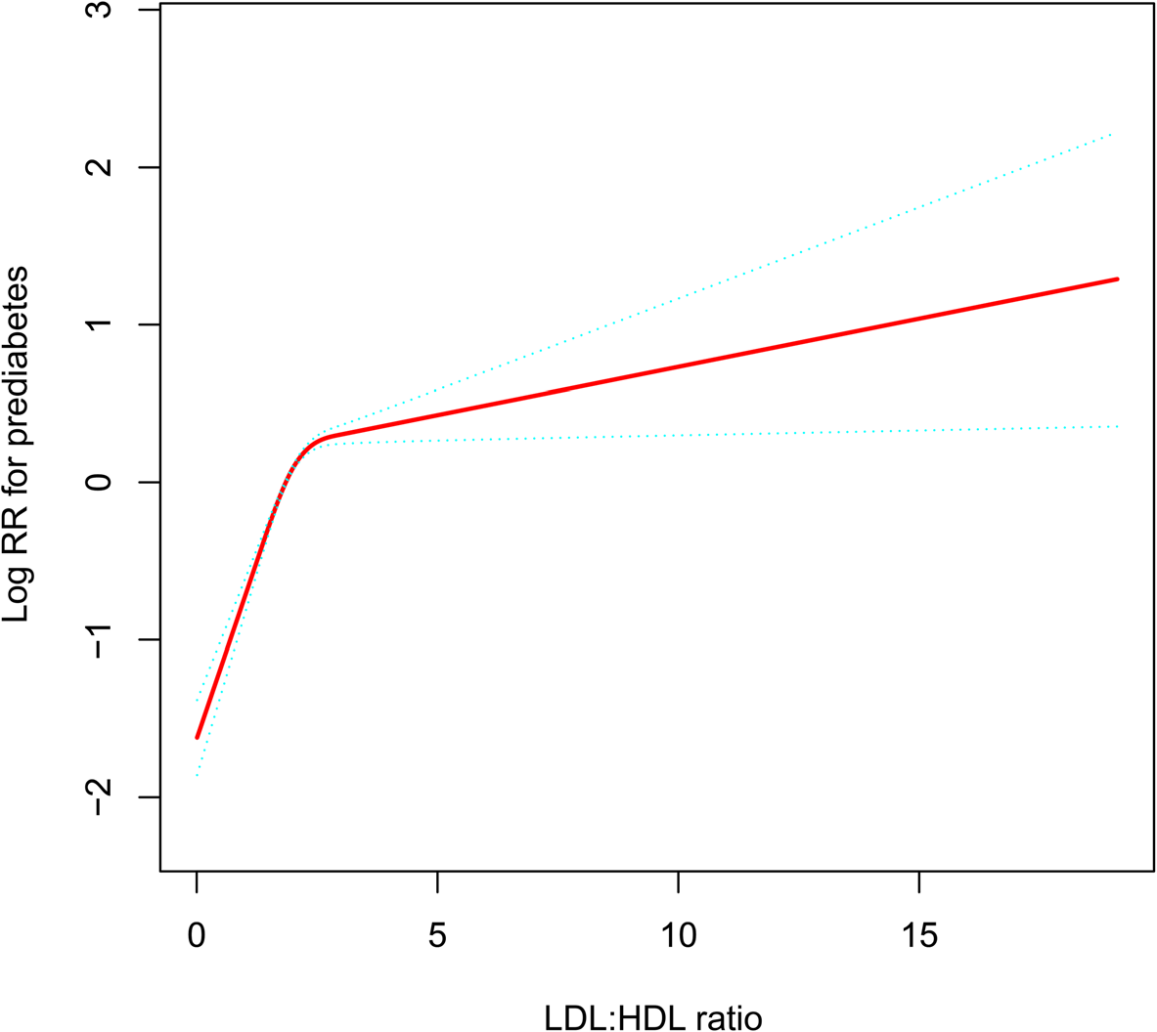
Hazard ratios (95% confidence intervals) for the non-linear relationship between LDL:HDL ratio and the risk of prediabetes. LDL:HDL ratio: low-density lipoprotein:high-density lipoprotein cholesterol ratio

Table 2 summarizes the relationship between LDL:HDL ratio and the incidence of prediabetes in the multivariate Cox regression analysis when used as a continuous and categorical variable, respectively. In the collinearity diagnostics of all covariates before establishing the multivariate Cox regression model, the variance inflation factor values of weight and LDL-C were greater than 5, which were considered to have high collinearity and were not included as covariates in the subsequent model (Supplementary Table 1). In the three Cox regression models with varying degrees of adjustment, whether the LDL:HDL ratio was computed as a categorical or continuous variable, the main results of all models were consistent. The LDL:HDL ratio was associated with prediabetes in the crude model (HR 1.15, 95% CI: 1.12, 1.17), and model 1 (HR 1.06, 95% CI: 1.01, 1.10), model 2 (HR 1.09, 95% CI: 1.04, 1.14) and model 3 (HR 1.09, 95% CI: 1.04, 1.15), respectively. The HR value was also only marginally attenuated in fully adjusted model 3, where a one-unit increase in the LDL:HDL ratio was associated with a 9% increased risk of prediabetes. After the LDL:HDL ratio was treated as a categorical variable, the Q1 category was designated as the reference category, and the risk of developing prediabetes progressively increased as the ratio of LDL:HDL elevated in the quartiles (*P* for trend < 0.0001). Additionally, it is also worth noting that the relationship of LDL:HDL ratio with prediabetes may be nonlinear after fitting by smoothing splines (Fig. 3). In summary, we can observe in all models that the LDL:HDL ratio was positively correlated with the new-onset prediabetes, and thus the LDL:HDL ratio is an independent risk factor for prediabetes.

**Table 2:** Cox regression analyses for the association between LDL:HDL ratio and the incidence of prediabetes

	Hazard ratios (95% confidence interval)			
	Crude model	Model 1	Model 2	Model 3
LDL:HDL ratio	1.15 (1.12, 1.17)	1.06 (1.01, 1.10)	1.09 (1.04, 1.14)	1.09 (1.04, 1.15)
LDL:HDL ratio (Quartile)				
Quartile 1	Ref	Ref	Ref	Ref
Quartile 2	1.50 (1.42, 1.58)	1.52 (1.42, 1.62)	1.46 (1.37, 1.55)	1.45 (1.35, 1.54)
Quartile 3	1.69 (1.61, 1.79)	1.76 (1.64, 1.90)	1.61 (1.50, 1.74)	1.58 (1.46, 1.70)
Quartile 4	1.62 (1.53, 1.70)	1.78 (1.61, 1.96)	1.61 (1.46, 1.78)	1.57 (1.42, 1.73)
*P*-trend	< 0.0001	< 0.0001	< 0.0001	< 0.0001

*Abbreviations*: *LDL:HDL ratio* low-density lipoprotein:high-density lipoprotein cholesterol ratio

Model 1 adjusted for FPG, TC, TG and HDL-C

Model 2 adjusted for FPG, TC, TG, HDL-C, age, sex and BMI

Model 3 adjusted for sex, age, BMI, SBP, DBP, FPG, TC, TG, HDL-C, ALT, BUN and Cr

### Sensitivity analysis

In several sensitivity analyses that excluded subjects with a family history of DM, subjects with abnormal systolic or diastolic blood pressure, subjects with normal BMI values, and subjects included according to the WHO’s diagnostic criteria for prediabetes, we obtained results that were consistent with the main analysis (Table 3).

**Table 3:** Adjusted hazard ratios and 95% confidence intervals for prediabetes risk associated with the LDL:HDL ratio in different test populations: sensitivity analysis

		LDL:HDL ratio quartiles					
	No.of subjects	LDL:HDL ratio	Q1	Q2	Q3	Q4	*P*-trend
Sensitivity-1	110,838	1.09 (1.01, 1.18)	Ref	1.34 (1.19, 1.50)	1.44 (1.27, 1.64)	1.48 (1.26, 1.74)	< 0.0001
Sensitivity-2	95,517	1.09 (1.04, 1.14)	Ref	1.45 (1.36, 1.55)	1.58 (1.47, 1.71)	1.57 (1.41, 1.73)	< 0.0001
Sensitivity-3	85,186	1.07 (1.01, 1.13)	Ref	1.45 (1.35, 1.57)	1.58 (1.45, 1.73)	1.58 (1.41, 1.77)	< 0.0001
Sensitivity-4	35,342	1.23 (1.14, 1.32)	Ref	1.34 (1.22, 1.48)	1.52 (1.37, 1.69)	1.62 (1.41, 1.86)	< 0.0001

*Abbreviations*: *LDL:HDL ratio* low-density lipoprotein:high-density lipoprotein cholesterol ratio

Note 1 (1) Sensitivity-1: including 110,838 subjects according to WHO’s diagnostic criteria for prediabetes; (2) sensitivity-2: excluding subjects with a family history of diabetes (*n* = 95,517); (3) sensitivity-3: excluding subjects whose SBP ≥ 140 mmHg or DBP ≥ 90 mmHg (*n* = 85,186); (4) sensitivity-4: excluding subjects with normal body mass index

Note 2 Adjusted for sex, age, BMI, SBP, DBP, FPG, TC, TG, HDL-C, ALT, BUN and Cr

### Stratified analyses

We conducted several stratified analyses by family history of DM, BMI, TG, age, and sex to explore other risk factors and possible special populations that influenced the relationship of LDL:HDL ratio with prediabetes (Table 4). The above parameters were stratified by clinical cut-off point. Among these subgroups, the relationship of prediabetes with the LDL:HDL ratio weakened with increased age and BMI, and in women and those with a family history of DM were more significantly associated. However, the effect of different TG levels on the risk of prediabetes associated with LDL:HDL ratio was not statistically different.

**Table 4:** Stratified association between LDL:HDL ratio and prediabetes by age, sex, family history of diabetes and BMI

Subgroup	No. of cases	unadjusted HR (95%CI)	adjusted HR (95%CI)	*P-*interaction
Age (years)				0.0018
20–29	563 (5.32%)	1.27 (1.14, 1.42)	1.21 (1.06, 1.38)	
30–39	3141 (8.11%)	1.20 (1.15, 1.25)	1.14 (1.07, 1.21)	
40–49	2888 (12.30%)	1.15 (1.10, 1.20)	1.14 (1.07, 1.21)	
50–59	2783 (18.16%)	1.00 (0.95, 1.05)	1.07 (1.01, 1.14)	
60–69	2053 (23.32%)	0.87 (0.82, 0.93)	1.00 (0.92, 1.08)	
≥ 70	924 (27.30%)	0.94 (0.86, 1.04)	1.04 (0.93, 1.16)	
Sex				< 0.0001
Men	7735 (14.84%)	0.98 (0.95, 1.00)	1.02 (0.97, 1.07)	
Women	4617 (9.58%)	1.23 (1.20, 1.26)	1.17 (1.12, 1.22)	
Family history of diabetes				0.0066
Yes	310 (14.04%)	1.34 (1.18, 1.53)	1.32 (1.15, 1.51)	
No	12,042 (12.28%)	1.14 (1.12, 1.17)	1.09 (1.04, 1.14)	
BMI (kg/m^2^)				< 0.0001
< 18.5	288 (5.04%)	1.32 (1.14, 1.53)	1.26 (1.08, 1.47)	
18.5–23.9	5399 (9.44%)	1.16 (1.13, 1.19)	1.15 (1.10, 1.20)	
24–27.9	5080 (16.94%)	0.92 (0.89, 0.96)	1.03 (0.98, 1.09)	
≥ 28	1585 (21.44%)	0.81 (0.76, 0.87)	0.96 (0.89, 1.03)	
TG (mmol/L)				0.9010
< 1.7	8276 (10.52%)	1.16 (1.13, 1.19)	1.06 (0.99, 1.13)	
≥ 1.7	4076 (18.82%)	1.06 (0.99, 1.13)	1.07 (0.99, 1.15)	

*Abbreviations*: *HR* hazard ratios, other abbreviations as in Table 1

*Note*: Models adjusted for the same covariates as in model 3 (Table 2), except for the stratification variable

### Evaluate the accuracy of LDL:HDL ratio in predicting prediabetes

Table 5 shows the AUROC of LDL:HDL ratio, HDL-C and LDL-C for prediabetes risk prediction. The AUROC of LDL:HDL ratio was larger compared to HDL-C and LDL-C (all Delong *P* < 0.0001), and the optimal threshold point for predicting the risk of prediabetes was 1.8759.

**Table 5:** Areas under the receiver operating characteristic curves for HDL-C, LDL-C, and LDL:HDL ratio in identifying prediabetes

	AUROC	95% confidence interval	Best threshold	Specificity	Sensitivity
HDL-C	0.5392^*^	0.5339–0.5446	1.3750	0.4769	0.5782
LDL-C	0.5500^*^	0.5446–0.5554	2.7550	0.5590	0.5139
LDL:HDL ratio	0.5715	0.5663–0.5767	1.8759	0.4552	0.6601

*Abbreviations*: *AUROC* area under the receiver operating curve, other abbreviations as in Table 1. *P < 0.0001, compared with LDL:HDL ratio

## Discussion

In this large longitudinal cohort study based on a Chinese adult population, we revealed for the first time a positive correlation of the LDL:HDL ratio with the risk of prediabetes. Sensitivity analysis results further confirmed that this association was very robust.

Prediabetes is the pathological stage that must go through to develop from normal blood glucose levels to type 2 DM and it is estimated that the annual conversion rate of prediabetes to DM is 5–10% [6]. IR and islet β-cell dysfunction are the pathological basis of the occurrence and development of prediabetes, and IR is also an important cause of “diabetic dyslipidemia” in patients with prediabetes and DM [10, 22, 23]. In prediabetic patients, dyslipidemia is usually manifested as elevated levels of LDL-C, TC, TG, and reduced HDL-C levels [22, 24]; among them, abnormally elevated LDL-C and decreased HDL-C levels will damage the function of pancreatic islet β cells to further aggravate IR, which will form a vicious cycle, and accelerate the progression from prediabetes to type 2 DM [25]. A series of clinical studies that have been completed have also confirmed that both LDL-C and HDL-C were important lipid parameters associated with the risk of glycemic metabolism [26–28]. In a recent study, Pan et al. using a two-sample Mendelian randomization analysis demonstrated that elevated LDL-C levels can significantly increase the risk of developing type 2 DM [27]; while in the study of the Chinese hypertensive population by Liu et al., it was found a U-shaped correlation between the level of LDL-C and the incidence of DM [26]. In contrast to LDL-C, HDL-C was inversely associated with the risk of glycemic metabolism. According to Drew BG et al., in their recent double-blind, placebo-controlled clinical trial, they recruited 13 patients with type 2 DM, and each patient was given intravenous injections of recombinant high-density lipoprotein (rHDL) and placebo saline at different times, with a 4-week interval between the two injections; the results showed that compared with a placebo injection, subjects injected with rHDL had significantly higher plasma HDL levels as well as apolipoprotein AI (ApoA-I) levels and plasma insulin levels, significantly enhanced β-cell function, while FPG levels decreased significantly. In further studies, the researchers found that HDL and ApoA-I can reduce FPG levels by activating AMP-activated protein kinase in skeletal muscle and enhancing the ability of skeletal muscle cells to uptake glucose [28]. In addition, studies have shown that in patients with type 2 DM, infusion of cholesteryl ester transfer protein inhibitor significantly reduced blood glucose levels by indirectly increasing plasma HDL levels [29, 30]. The above studies have shown that HDL-C is a favorable factor for blood glucose metabolism and can have a positive impact on patients with glucose metabolism disorders.

Recently, some researchers have found that the LDL:HDL ratio, a combined index which is calculated as LDL-C divided by HDL-C, was also closely related to DM, NAFLD, and many other metabolic-related diseases, and this parameter was also regarded as a potential IR surrogate marker [15–17, 31–33]. However, the relationship of LDL:HDL ratio with the incidence of prediabetes has not been clarified. This study found that the LDL:HDL ratio was an independent risk factor for prediabetes. To our knowledge, our research first revealed the relationship of LDL:HDL ratio with prediabetes, a finding that may provide a valuable reference for the primary prevention of prediabetes.

The underlying mechanism of the relationship of LDL:HDL ratio with prediabetes remains unclear, and there are several possible explanations. In prediabetic patients, a high LDL:HDL ratio is usually caused by normal or elevated plasma LDL-C levels and significantly decreased plasma HDL-C levels [22]. The elevated LDL-C levels may exert potential prediabetic effects by reducing both the maximum glucose-stimulated insulin secretion and the basal proliferation of human islet β-cells; whereas reduced HDL-C levels could weaken its protective effect on islet β-cells apoptosis, resulting in lower islet β-cell numbers and insulin secretion; the occurrence and development of prediabetes may be the outcome of the combined action of the two [25]. Moreover, from Table 1 we can find that people with a high LDL:HDL ratio generally have higher TG levels; previous studies have shown that elevated TG levels in skeletal muscle increased skeletal muscle IR and systemic IR, which means that high TG levels may further promote the occurrence of prediabetes [34]. We also further evaluated the role of TG in LDL:HDL ratio-related prediabetes risk in subgroup analysis, and the results showed that no significant interaction was found, and further studies are needed to explore the potential association.

Some interesting results were also found in the stratified analysis of the current study. The LDL:HDL ratio-related prediabetes risk diminished with increasing age and BMI, and the mechanisms behind this age- and BMI-related effect heterogeneity may be explained in several ways. On the one hand, age-associated increases in IR and the number of comorbidities, as well as the decline in general health, may result in a relatively weakened association of the LDL:HDL ratio with prediabetes [35]; with the increase of BMI, the IR will be significantly enhanced, making overweight and even obesity a major risk factor for prediabetes [36]. On the other hand, poor diet and lifestyle may increase prediabetes risk associated with LDL:HDL ratio in younger age groups [37]; the risk of prediabetes associated with LDL:HDL ratio is higher in non-obese people with lower BMI may be attributed to ectopic fat deposition, insulin secretion disorders, low birth weight due to uterine malnutrition, and epigenetic changes in the human genome, which can lead to severe glucose metabolism disorders and more prevalent in Asian population [38]. At the same time, we found that in the sex subgroups women had a significantly higher LDL:HDL ratio-related prediabetes risk, which was confusing. Because in the current study, there was a significantly higher proportion of women than men in the low LDL:HDL ratio categories, and the incidence of prediabetes in women was found to be lower than in men in the sex subgroups (9.58% vs 14.84%), therefore it is oddly that women showed higher LDL:HDL ratio-related risk of prediabetes than men (*P*-interaction < 0.0001). The reason for this phenomenon may be suggested by the age subgroups in the stratified analysis. We can find that about 70% of all subjects with new-onset prediabetes are older than 50 years old, which indicated that prediabetes in most women occurred after menopause [39]. Based on some available evidence, we speculate that the increased risk of prediabetes in postmenopausal women may be associated with changes in ovarian aging-related hormone levels. It is well known that postmenopausal women experience accelerated ovarian aging, markedly decreased estrogen levels, and markedly increased androgen levels; the most immediate effects of these changes are further increased adipose tissue mass, fat redistribution, decreased skeletal muscle mass, and decreased insulin sensitivity [40]; most notable of which is the redistribution of body fat in women after menopause. According to existing studies, estrogen and androgen play different roles in fat deposition, among which estrogen is mainly related to peripheral fat storage in the subcutaneous region of the buttocks and femur, while androgen is mainly related to the accumulation of visceral abdominal fat [41]. In postmenopausal women, the levels of sex hormones in the body are significantly changed, and the ratio of androgen to estradiol is significantly increased, which may be an important reason for central fat accumulation, visceral fat increase, and abdominal obesity [40, 42]. These deleterious patterns of fat deposition all significantly increase the risk of prediabetes in postmenopausal women. In the subgroup of family history of DM, those with a family history of DM showed a higher risk of LDL:HDL ratio-related prediabetes. This discrepancy may be because the occurrence of DM is influenced by genetic factors, and only a single gene mutation such as PPARgamma, ATK2, and insulin receptor gene can cause severe IR [43]. Furthermore, there was a lot of evidence that the adiponectin gene, HFE hereditary hemochromatosis gene, etc. may also affect the susceptibility to type 2 DM; individuals with the susceptibility genes were more likely to develop prediabetes if environmental factors altered the expression of these genes [44].

### Strengths and limitations

This study has some notable merits: (1) This is the first study to explore the relationship between LDL:HDL ratio and prediabetes, several special populations were found in the stratified analyses, which can provide a new strategy for the accurate screening of prediabetes as well as the prevention and intervention of DM. (2) The study population comes from multiple regions in China, with a huge number and a wide age distribution, which is well representative of the Chinese population. (3) Strict statistical analysis was performed in this study, and multiple sensitivity analyses and subgroup analyses were conducted. The positive correlation of the LDL:HDL ratio with the incidence of prediabetes is stable among all populations, so the conclusions of this research are relatively reliable.

Of course, this study also has some limitations: (1) As the diagnostic criteria for prediabetes recommended by ADA were adopted in the current study [12], its relatively low diagnostic threshold for IFG may include more patients with new-onset prediabetes. Nevertheless, we applied the WHO-recommended diagnostic criteria for prediabetes in the sensitivity analysis, based on a higher diagnostic threshold of IFG (FPG: 6.1–6.9 mmol/L) [21], and also obtained results consistent with the main analysis. (2) The diagnosis of prediabetes in the present study was only based on FPG levels, which may cause us to miss some new cases of prediabetes. However, we still demonstrated a significant correlation of LDL:HDL ratio with prediabetes in a smaller number of cases. (3) As the study design of the retrospective cohort, even if we adjusted for a large number of confounders, residual confounding due to measurement error in the assessment of confounders and the unavailability of data for some unmeasured factors could not be ruled out. (4) The results obtained in this study are based on a Chinese population cohort, most of which are from cities in southern China, so our results are more suitable for generalization in southern Chinese populations, while further research is needed in northern populations and non-Chinese populations. (5) Since this study did not distinguish between different types of HDL-C, the effect of dysfunctional HDL-C on blood glucose metabolism could not be excluded, which may affect the correlation between LDL:HDL ratio and prediabetes, and further analyses of the different roles of HDL-C and its subpopulations are required in future studies [45].

## Conclusion

All in all, the LDL:HDL ratio is an independent risk factor for prediabetes in the Chinese adult population. An increased LDL:HDL ratio is positively correlated with the risk of prediabetes, and this positive relationship is stable across all tested populations. It is also worth noting that high LDL:HDL ratios are associated with a higher risk of prediabetes in young adults, women, those with a family history of DM, and non-obese individuals. These results indicate that the LDL:HDL ratio may be one of the lipid-lowering targets for the prevention and treatment of prediabetes.

## Supplementary Information

**Additional file 1.**

## Data Availability

The dataset supporting the conclusions of this article is available in the DRYAD repository, [unique persistent identifier and hyperlink to dataset in 10.5061/dryad.ft8750v].

## Abbreviations

LDL:HDL ratio: Low-density lipoprotein:high-density lipoprotein cholesterol ratio
IR: Insulin resistance
ADA: American Diabetes Association
WHO: World Health Organization
BMI: Body mass index
DM: Diabetes mellitus
IFG: Impaired fasting glucose
FPG: Fasting plasma glucose
NAFLD: Nonalcoholic fatty liver disease
TC: Total cholesterol
TG: Triglyceride
HDL-C: High-density lipoprotein cholesterol
LDL-C: Low-density lipoprotein cholesterol
ALT: Alanine aminotransferase
AST: Aspartate aminotransferase
BUN: Blood urea nitrogen
Cr: Creatinine
HR: Hazard ratios
CI: Confidence intervals
SBP: Systolic blood pressure
DBP: Diastolic blood pressure
AUROC: area under the receiver operating curve
rHDL: recombinant high-density lipoprotein
