# Supplementary material for: Association of LDL:HDL ratio with prediabetes risk: a longitudinal observational study based on Chinese adults
Source: Lipids Health Dis. 2022 May 15;21:44. doi: 10.1186/s12944-022-01655-5 (PMC9107720; doi:10.1186/s12944-022-01655-5)
Supplement: Supplementary file 1 — Additional file 1. [file 12944_2022_1655_MOESM1_ESM.docx]

Supplementary Table 1: Collinearity diagnostics steps.

|  | Variance inflation factor | | |
| --- | --- | --- | --- |
|  | Step 1 | Step 2 | Step 3 |
| LDL:HDL ratio | 10.2 | 10.2 | 5.3 |
| Sex | 3.3 | 3.2 | 3.2 |
| Age | 1.4 | 1.4 | 1.4 |
| Height | 53 | 2.1 | 2.1 |
| Weight | 168.3 | NA | NA |
| BMI | 97.6 | 1.5 | 1.5 |
| SBP | 2.1 | 2.1 | 2.1 |
| DBP | 2 | 2 | 2 |
| FPG | 1 | 1 | 1 |
| TC | 7.3 | 7.3 | 4.4 |
| TG | 1.6 | 1.6 | 1.5 |
| HDL-C | 6 | 6 | 4.7 |
| LDL-C | 11.5 | 11.5 | NA |
| ALT | 3.3 | 3.3 | 3.3 |
| AST | 2.9 | 2.9 | 2.9 |
| BUN | 1.2 | 1.2 | 1.2 |
| Cr | 2.1 | 2.1 | 2.1 |
| Family history of diabetes | 1 | 1 | 1 |
| Smoking status | 3.1 | 3.1 | 3.1 |
| Drinking status | 3.1 | 3.1 | 3.1 |

Note-1: Variance inflation factor = 1/(1-R^2^). Abbreviations as in Table 1.

Note-2: The variables with Variance inflation factor >5 will be regarded as collinear variables and cannot be included in the multiple regression model.
